# Cell-Free DNA: Hope and Potential Application in Cancer

**DOI:** 10.3389/fcell.2021.639233

**Published:** 2021-02-22

**Authors:** Yan-yan Yan, Qiao-ru Guo, Feng-hua Wang, Rameshwar Adhikari, Zhuang-yan Zhu, Hai-yan Zhang, Wen-min Zhou, Hua Yu, Jing-quan Li, Jian-ye Zhang

**Affiliations:** ^1^School of Medicine, Shanxi Datong University, Datong, China; ^2^Key Laboratory of Molecular Target and Clinical Pharmacology and the State Key Laboratory of Respiratory Disease, School of Pharmaceutical Sciences and the Fifth Affiliated Hospital, Guangzhou Medical University, Guangzhou, China; ^3^The First Affiliated Hospital, Hainan Medical University, Haikou, China; ^4^Guangzhou Institute of Pediatrics/Guangzhou Women and Children's Medical Center, Guangzhou Medical University, Guangzhou, China; ^5^Research Centre for Applied Science and Technology, Tribhuvan University, Kirtipur, Nepal; ^6^State Key Laboratory of Quality Research in Chinese Medicine, Institute of Chinese Medical Sciences, Avenida da Universidade, Taipa, China

**Keywords:** cell-free DNA (cfDNA), cancer, diagnosis, therapeutic effect evaluation, liquid biopsy

## Abstract

Cell-free DNA (cfDNA) is easily accessible in peripheral blood and can be used as biomarkers for cancer diagnostics, prognostics, and therapeutics. The applications of cfDNA in various areas of cancer management are attracting attention. In this review article, we discuss the potential relevance of using cfDNA analysis in clinical oncology, particularly in cancer screening, early diagnosis, therapeutic evaluation, monitoring disease progression; and determining disease prognosis.

## Introduction

Cell-free DNA (cfDNA) is released from cells into the circulatory system throughout the body. It was first discovered by Mandel and Métais in 1948 (Mandel and Metais, 1948). cfDNA can be found in plasma (Mandel and Metais, 1948) and other body fluids such as, cerebral spinal fluid (CSF) (Rhodes et al., 1994), pleural fluid (Sriram et al., 2012), urine (Sidransky et al., 1991; Zhang et al., 1999), saliva (Mithani et al., 2007; Wang et al., 2015), and others. Previous studies indicated that most of the plasma cfDNA molecules originate from the hematopoietic system in healthy individuals (Lui et al., 2002; Sun et al., 2015). However, in certain physiological or pathological conditions, such as pregnancy, organ transplantation, and cancers, the related/affected tissues could release additional DNA into peripheral circulation (Leon et al., 1977; Lo et al., 1997, 1998). Therefore, detection of cfDNA in peripheral blood could identify abnormalities of individuals in a noninvasive manner. In recent years, a variety of technologies have emerged based on the analysis of cfDNA for noninvasive prenatal testing (NIPT) (Lo et al., 1997, 2007; Hyett et al., 2005; Wong and Lo, 2015; Hudecova and Chiu, 2017; Bianchi and Chiu, 2018; Malan et al., 2018; Vivanti et al., 2019; Zhang J. et al., 2019), monitoring organ transplantation (Lo et al., 1998; Gielis et al., 2015; Gala-Lopez et al., 2018; Sherwood and Weimer, 2018), and detecting immune diseases (Zhang et al., 2014; Beranek et al., 2017; Dunaeva et al., 2018; Xu et al., 2018; Duvvuri and Lood, 2019), as well as cancers.

Analysis of protein biomarkers and nucleic acids has become very popular for cancer diagnosis (Huang et al., 2020), risk stratification, and molecular targeting therapeutics (Zhang D. et al., 2019; Wang et al., 2020). However, many biomarker studies are based on analysis of tumor tissues that are obtained from invasive surgical procedures and may not be accessible in some cases. New approaches with noninvasive procedure are urgently needed. cfDNA analysis has attracted increasing attention because of its easy accessibility, noninvasive nature, and potential tumor specificity through quantitative detection or specific sequencing. In Table 1, we summarized several cfDNA applications in different diseases. Therefore, considerable efforts have been made to explore the potential application of cfDNA in clinical cancer management. The sensitivity of cfDNA analytic technologies has been greatly improved due to the advancement of molecular biology and next-generation sequencing (NGS) approaches. cfDNA analysis is widely used in various areas of cancer diagnostics and prognostics, as well as cancer drug resistance, and early screening.

**Table 1:** Summary of cfDNA applications in different diseases.

**Disease**	**Marker(s)**	**Technical method**	**Clinical significance**	**Sample**	**References**
Non-invasive prenatal testing (NIPT)	Fetal DNA	PCR, sequencing; Y-PCR assay	Trisomy 13, 18, or 21; fetal achondroplasia; dominant monogenic diseases; thalassemias; determination of fetal sex	Maternal plasma or serum	Lo et al., 1997; Wong and Lo, 2015; Hudecova and Chiu, 2017; Bianchi and Chiu, 2018; Malan et al., 2018; Vivanti et al., 2019; Zhang J. et al., 2019
Organ transplantation	Donor-specific DNA	PCR, Sequencing	Monitoring organ transplantation	Plasma of transplant recipients	Lo et al., 1998; Gielis et al., 2015; Gala-Lopez et al., 2018; Sherwood and Weimer, 2018
Immune diseases	Elevated levels of cfDNA; LINE-1 hypermethylation	Picogreen Kit; qPCR (quantitative real-time PCR); methylation-specific quantitative PCR, sequencing	Detecting and assessing systemic lupus erythematosus (SLE) disease activity and monitoring treatment; exacerbated psoriasis; relapsing remitting multiple sclerosis; autoimmune rheumatic diseases	Plasma or serum of patients	Zhang et al., 2014; Beranek et al., 2017; Dunaeva et al., 2018; Xu et al., 2018; Duvvuri and Lood, 2019
Cancer	Circulating tumor DNA	PCR; sequencing	Diagnosis; predicting response to therapy; indicating prognosis	Plasma and serum of cancer patients	Wan et al., 2017; Wang and Xu, 2019; Fares et al., 2020

**Figure 1:**
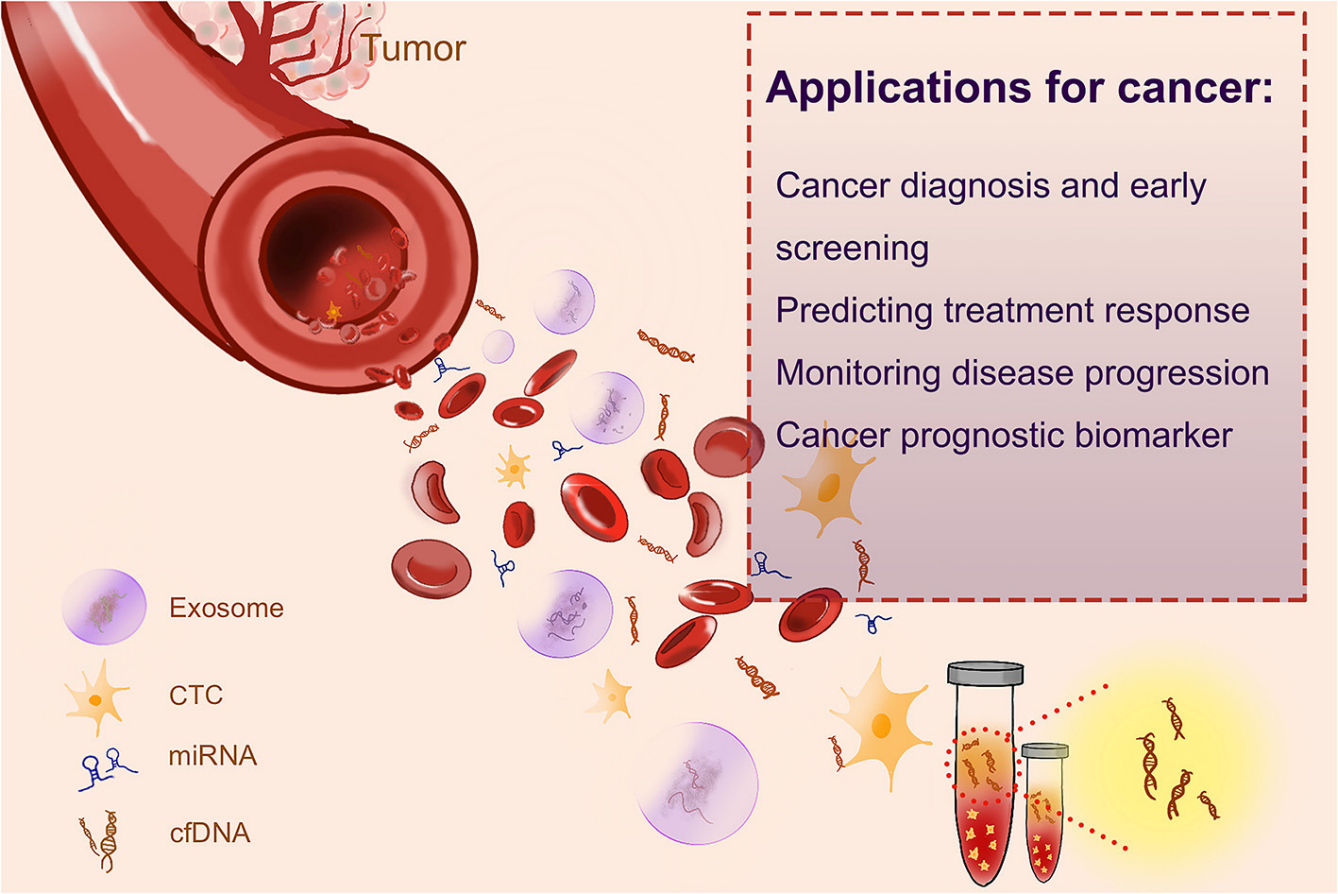
The applications of cfDNA in cancer.

Cancer patients usually have a high level of cfDNA in their serum or plasma as a result of cellular necrosis or apoptosis, because tumor cells divide faster than normal cells, and cfDNAs are released in a high proportion (Sorenson et al., 1994; Vasioukhin et al., 1994; Raja et al., 2018). The fraction of cfDNA that derived from tumor cells is named circulating tumor DNA (ctDNA) (Leon et al., 1977; Shu et al., 2017). In recent years, both cfDNA and ctDNA have gotten huge attention as novel blood biomarkers, as quantification and kinetic analysis of cfDNA (Diehl et al., 2008) and molecular profiling of ctDNA have suggested their predictive and prognostic values (Iizuka et al., 2006; Tokuhisa et al., 2007). Several liquid biopsy tests, designed for the identification of cancer-specific mutations, have been recommended as companion diagnostic (CDx) tests, by the European Medicines Agency (EMA) and Food and Drug Administration (FDA) of USA, to guide therapeutic decision making. Such tests include the cobas EGFR Mutation Test for non-small lung cancer or BRAC Analysis CDx for breast and ovarian cancer. Epi proColon^®^, based on the analyses of the methylation status of the SEPT9 gene, is the first and only FDA-approved blood-based test for the detection of colorectal cancer. The applications of cfDNA in cancer are shown in Figure 1.

## cfDNA for Cancer Diagnosis and Early Screening

Analysis of cfDNA has become a promising noninvasive approach in cancer diagnosis (Haber and Velculescu, 2014; Siravegna et al., 2017). The applications of cfDNA-based cancer liquid biopsy in recent year are summarized in Table 2. cfDNA comes from fragmented DNA released by cells into the circulation, most commonly as a result of cell death. In healthy individuals, the blood cfDNA originates from germline DNA released by normal cells. In cancer patients, a portion of cfDNA comes from tumor cells, named circulating-tumor DNA (ctDNA), which may contain tumor-specific variations corresponding to the patient's tumor, such as mutated tumor suppressor genes or oncogenes (Wang et al., 2004), microsatellite instability (MSI) (Shaw et al., 2000), and DNA methylation (Fujiwara et al., 2005). Based on some quantitative studies, it is found that the concentration of cfDNA in healthy subjects is between 0 and 100 ng/ml of blood with an average of 30 ng/ml, whereas the concentration of cfDNA in the blood of cancer patients varies from 0 to 1,000 ng/ml, with an average of 180 ng/ml (Leon et al., 1977; Esposito et al., 2017; Phallen et al., 2017).

**Table 2:** Summary of cfDNA-based cancer liquid biopsy studies.

**Cancer type**	**Marker(s)**	**Technical method**	**Clinical significance**	**Sample**	**References**
Hematological malignancies	Copies of circulating NPM mutation A (NPM mut.A) DNA; TP53 mutations; MYD88 p.L265P mutation	RT-PCR and sequencing analysis; direct sequencing and a PCR-restriction digestion analysis (RFLP); droplet digital PCR (ddPCR)	Diagnosis	Plasma; serum; cerebrospinal fluid (CSF)	Hosny et al., 2009; Quan et al., 2015; Zorofchian et al., 2018
Thyroid cancer	Cell-free DNA quantity and integrity index 180/67	Quantitative real-time PCR (qPCR)	Diagnosis	Plasma	Salvianti et al., 2017
Colorectal cancer	ALU115; dozens of DNA hypermethylation markers (e.g., SEPT9 and IKZF1, EMBP1, KCNQ5, CHST11, APBB1IP, and TJP2)	qPCR; methylated CpG tandem amplification and sequencing (MCTA-Seq)	Diagnosis	Plasma	Bhangu et al., 2017; Li et al., 2019
	High cfDNA and ctDNA levels; methylated circulating DNA biomarkers (EYA4, GRIA4, ITGA4, MAP3K14-AS1, MSC)	ddPCR	Predicting response to therapy	Plasma	Barault et al., 2018; Lyskjaer et al., 2019
	High cfDNA levels; methylation levels of HLTF, HPP1/TPEF, hMLH1, TAC1, and SEPT9	ddPCR; quantitative methylation-specific PCR	Indicating prognosis	Plasma; serum	Wallner et al., 2006; Tham et al., 2014; Hamfjord et al., 2019
Gastric cancer	Mutations of cadherin (CDH1), phosphatidylinositol-4,5-bisphosphate 3-kinase catalytic subunit alpha (PIK3CA), ARID1A (AT-rich interactive domain 1A), epidermal growth factor receptor (EGFR), and phosphatase and tensin homolog deleted on chromosome 10 (PTEN); 5-hydroxymethylcytosine (5hmC)	Bidirectional sequencing; 5 hmC quantification	Diagnosis	Plasma	Samuels et al., 2004; Li et al., 2005, 2017; Velho et al., 2005; Kaurah et al., 2007; Heald et al., 2010; Wen et al., 2010; Corso et al., 2011, 2012; Liu et al., 2011; Lee et al., 2012; Zang et al., 2012; Chen et al., 2013
Hepatocellular carcinoma	RASSF1A promoter hypermethylation	Methylation-specific PCR	Diagnosis	Serum	Chan et al., 2008
	Post-radiotherapy (RT) cfDNA levels; mutation of BAX, CYP2B6 and HNF1A	RT-PCR; NGS	Predicting response to therapy	Plasma	Park et al., 2018; Alunni-Fabbroni et al., 2019
	Methylation of insulin-like growth factor-binding protein 7 (IGFBP7); higher level of circulating DNA	RT-PCR	Indicating prognosis	Serum; plasma	Ren et al., 2006; Li et al., 2018
HPV-positive metastatic cervical cancer	HPV cfDNA	Duplex digital droplet PCR (ddPCR)	Diagnosis; predicting response to therapy	Serum	Kang et al., 2017
Nasopharyngeal carcinoma	Epstein–Barr virus (EBV) DNA	PCR	Diagnosis	Plasma	Chan et al., 2017
Lung cancer	act-EGFR mutant allele frequency (MAF) and T790M/act-EGFR MAF ratio; 36 cancer-related genes; TP53, RB1, BRAF, KIT, NOTCH1–4, PIK3CA, PTEN, FGFR1, MYC, MYCL1, and MYCN	ddPCR; NGS	Predicting response to therapy	Plasma	Almodovar et al., 2018; Del Re et al., 2018; Guibert et al., 2019
	EGFR19del, L858R, and T790M; cfDNA concentration; KRAS mutation	ddPCR; RFLP-PCR; mutant-enriched PCR	Indicating prognosis	Plasma or serum	Ai et al., 2016; Yanagita et al., 2016
Prostate cancer	Androgen receptor gene (AR) copy numbers (CN) and mutations; cfDNA concentration and mutations (BRCA2, PALB2); AR amplification, TMPRSS2-ERG fusion, PTEN gene deletion, NOTCH1 locus amplification along with genomic amplifications	dPCR and target sequencing; targeted cfDNA sequencing; whole-genome sequencing (WGS)	Predicting response to therapy	Plasma, urine	Xia et al., 2016; Goodall et al., 2017; Sumiyoshi et al., 2019
	Hypermethylation patterns of two genes (GSTP1 and APC)	Methylation-specific PCR	Indicating prognosis	Plasma	Hendriks et al., 2018
Breast cancer	ESR1 mutation	Digital PCR	Predicting response to therapy	Plasma	Beije et al., 2018
	cfDNA concentration and cfDNA integrity (cfDI); ALU-247, ALU-115, and cfDNA Integrity; methylation of KLK10, SOX17, WNT5A, MSH2, GATA3	RT-qPCR; quantitative methylation-specific PCR	Indicating prognosis	Plasma	Cheng et al., 2018; Hussein et al., 2019; Panagopoulou et al., 2019
Cervical cancer	Increased cfDNA allele fraction deviation (AFD)	Targeted deep sequencing	Predicting response to therapy	Plasma	Tian et al., 2019
Bladder carcinoma	Presence and dynamics of ctDNA	Ultra-deep multiplex polymerase chain reaction–based next-generation sequencing	Predicting response to therapy	Plasma	Christensen et al., 2019
Pancreatic cancer	ABL1, ATM, DNMT3A, FLT3, HNF1A, NRAS, and SMAD4	NGS; Library Preparation Kit	Predicting response to therapy	Plasma	Vietsch et al., 2017
	Pretreatment cfDNA fragment size of ≤167 bp (mode) and high pre-treatment cfDNA levels	Agilent 2100 Bioanalyzer and the Agilent High Sensitivity DNA chip	Indicating prognosis	Plasma	Lapin et al., 2018
Esophageal cancer	Mutations of TP53, PIK3CA, ERBB2	NGS; ddPCR	Predicting response to therapy	Plasma	Pasternack et al., 2018
Oral squamous cell carcinoma	Higher cfDNA levels	Quantitative spectrometry	Indicating prognosis	Plasma	Lin et al., 2018
Metastatic melanoma	Baseline cfDNA concentration	ddPCR	Indicating prognosis	Plasma	Valpione et al., 2018

It is reported that the size (Kamat et al., 2006; Gorges et al., 2012) and stage (Nawroz et al., 1996; Bettegowda et al., 2014) of tumors are correlated with the blood level of ctDNA. There are 100–1,000 copies of ctDNA per 5 mL of blood in patients with stage IV or advanced tumors, and only 10 copies of ctDNA in those with early stage cancers (Bettegowda et al., 2014). Additional amount of ctDNA is also seen in patients with metastatic tumors. Quantification on patients with relapsed high-grade serous ovarian carcinoma (HGSOC) has indicated an increase of six copies of ctDNA per mL blood for an additional 1 cm^3^ of tumor (Parkinson et al., 2016).

### Hematological Malignancies

Some studies analyzed the cfDNA in hematologic malignancies. TP53 (G249 T, G249A, T176C, C250 T, and T238 G) and nucleophosmin mut.A (a duplication of the TCTG at positions 956–959) mutations were found in cfDNA from patients with acute myelogenous leukemia (AML) and non-Hodgkin's lymphoma (NHL) (Hosny et al., 2009; Quan et al., 2015). The diagnosis of lymphomas in the central nervous system (CNS) is complex, and obtaining brain biopsy is of high risk of complications. Biomarkers in blood and cerebrospinal fluid could be potential tools for early diagnosis of lymphomas. Zorofchian et al. examined a patient with suspected CNS lymphoma by detecting MYD88 mutation (L265 P and V217 F) in the cerebrospinal fluid (CSF). They suggested that analysis of cfDNA in CSF could be a minimally invasive diagnostic tool for CNS lymphomas (Zorofchian et al., 2018). Other studies also proved the potential of detecting MYD88 mutation in CSF as well as in plasma (Fontanilles et al., 2017; Hiemcke-Jiwa et al., 2019). Patients with lymphoma showed a higher level of cfDNA in their plasma than did healthy subjects. Hohaus et al. (2009) reported that the cfDNA level is different in patients with diffuse large B-cell lymphoma (DLBCL), mantle cell lymphoma (MCL), and Hodgkin's lymphoma (HL). Nucleosomal DNA (ncDNA) is another source of cfDNA. The changes of ncDNA corresponded with response to treatment, suggesting that ncDNA could be a valuable biomarker for hemopoietic cancer patients (Mueller et al., 2006). Taken together, cfDNA can be used for detecting gene mutation and chromosomal abnormalities. The level of blood cfDNA might serve as an important noninvasive diagnostic tool for patients with hemopoietic cancers.

### Thyroid Cancer

Salvianti et al. adopted a quantitative real-time PCR (qPCR) approach based on quantification of two amplicons with different lengths (67 and 180 bp) to evaluate the integrity index 180/67. They reported that the cfDNA integrity index 180/67 can monitor cfDNA fragmentation in thyroid cancer. Thus, the cfDNA integrity index 180/67 could be used as a circulating biomarker for the diagnosis of thyroid nodules. The quantity of cfDNA is higher in patients affected by nodular thyroid diseases than in healthy individuals. Importantly, the cfDNA integrity index was higher in patients with cytologically diagnosed thyroid carcinoma (Thy4/Thy5) than in individuals with benign nodules (Thy2) (Salvianti et al., 2017).

### Colorectal Cancer

It is reported that KRAS mutation can be identified using cfDNA. Wang et al. uncovered KRAS mutations (in codons 12, 13, and 61) in patients with stage I–IV colorectal cancer and found these mutations in blood samples. They showed that there are about 45% of concordance between the CRC tumor tissues and cfDNA. KRAS mutations are not found in cfDNA of healthy subjects (Wang et al., 2004). In the study of Anker et al. (1997), a high (86%) KRAS mutation in codon 12 was found in tumor tissue and blood. Thierry et al. (2014) reported an even higher (96%) mutation of KRAS in codons 12 and 13 in tumor tissues and blood, and the mutation of BRAF (V600E) reached 100%. Bhangu et al. (2017) found that the level of ALU115 in cfDNA could be an indicator of colorectal cancer (CRC).

Li et al. adopted a fully methylated molecule algorithm with the methylated CpG tandem amplification and sequencing (MCTA-Seq) method, to examine the blood sample of patients with CRC (*n* = 147) and healthy individuals (*n* = 136), as well as cancer and adjacent noncancerous tissues (*n* = 66). They found that a number of biomarkers of DNA hypermethylation including the known (e.g., SEPT9 and IKZF1) and the novel (e.g. TJP2, EMBP1, APBB1IP, CHST11, and KCNQ5) genes were detected in cfDNA of CRC (Li et al., 2019).

### Gastric Cancer

It was known that mutations in genes such as CDH1 (Kaurah et al., 2007; Corso et al., 2012; Chen et al., 2013), PIK3CA (Samuels et al., 2004; Li et al., 2005; Velho et al., 2005; Lee et al., 2012), ARID1A (AT-rich interactive domain 1A) (Zang et al., 2012), EGFR (Corso et al., 2011; Liu et al., 2011), and PTEN (Heald et al., 2010; Wen et al., 2010) existed in gastric cancer, providing potential circulating DNA for detection of gastric cancer. For example, a clinical correlation analysis of ctDNA in patients with gastric adenocarcinoma showed that PIK3CA is one of the most frequently affected character-altered alterations, which can help to indicate clinically relevant genomic data for clinical diagnosis and treatment (Maron et al., 2019). Li et al. (2017) reported that 5 hmC of circulating cfDNA could specifically and sensitively separate gastric cancer patients from healthy subjects.

### Hepatocellular Carcinoma

DNA methylation is involved in activating protooncogenes or inactivating tumor-suppressor genes. The study of Yeo et al. (2005) revealed that RASSF1A promoter hypermethylation in blood cfDNA is correlated with the size of hepatocellular carcinoma (HCC). Furthermore, patients with hypermethylated RASSF1A in cfDNA at the time of diagnosis or 1 year after tumor resection have shorter DFS (Chan et al., 2008). The detection of RASSF1A promoter methylation in cfDNA in the blood is 90% in HCC patients and only 10% in healthy subjects. The mean blood level of methylated RASSF1A in HCC patients is significantly higher than that in healthy individuals (Mohamed et al., 2012).

### Virus-associated Cancer

cfDNA analysis has been used to screen diseases before clinical onset (Mao et al., 1994; Gormally et al., 2006; Phallen et al., 2017). cfDNA analysis has shown to be an effective way to screen the asymptomatic early-stage nasopharyngeal carcinoma caused by Epstein–Barr virus (EBV), as the viral cfDNA level is much higher than that of ctDNA. One study involved in screening of >20,000 asymptomatic subjects for EBV DNA in blood has led to the diagnosis of nasopharyngeal carcinoma in 34 individuals (Chan et al., 2017). Identification of these early diagnosed patients improved the 3-year progression-free survival (Chan et al., 2017). Circulating cfDNA of human papillomavirus (HPV) (HPV ccfDNA) may be used as a unique tumor biomarker for HPV-associated malignancies, including cervical cancer (Kang et al., 2017).

## cfDNA in Monitoring Disease Progression and Predicting Treatment Response in Cancers

Since access to longitudinal tumor tissues are limited, some studies chose to focus on the characterization of cfDNA for rapid, noninvasive monitoring for disease progression, treatment response, and disease relapse.

### Hepatocellular Carcinoma

cfDNA is used as a prognostic biomarker in patients with advanced HCC. Park et al. examined the clinical significance of cfDNA in patients with HCC treated with radiotherapy (RT). They found that the level of post-RT cfDNA was negatively correlated with treatment outcome, indicating the potential of using post-RT cfDNA level as a predictor of treatment response and local control (LC) (Park et al., 2018). Alunni-Fabbroni et al. investigated the use of cfDNA and ctDNA in HCC patients to assess therapeutic response and clinical outcome. The level of cfDNA was shown to have a significant correlation with tumor metastases and patient survival. In addition, a dynamic study on cfDNA uncovered a trend of cfDNA level with the clinical history of patients, suggesting its usefulness as a biomarker for monitoring treatment response. Twenty-eight variants were identified in different combinations at different time points using NGS-based analysis on ctDNA. Among these variants, BAX, CYP2B6, and HNF1A genes showed the highest frequency of mutation and a significant association with the clinicopathological characteristics of patients, indicating their possible roles as driver genes in the clinical setting (Alunni-Fabbroni et al., 2019). The relationship between the level of blood cfDNA and clinicopathological characteristics was investigated in other studies. It was found that HCC patients with a larger tumor size (≥5 cm) or vascular invasion often showed higher levels of blood cfDNA (Iizuka et al., 2006; Huang et al., 2012). Huang et al. (2012) investigated the dynamic change of the level of blood cfDNA in patients with stage I–IV HCC and found that the level of cfDNA decreases after removal of tumors.

### Lung Cancer

The correlation between osimertinib treatment outcome and activating EGFR (act-EGFR) mutations and T790M in cfDNA was evaluated in patients with advanced non-small cell lung cancer (NSCLC). Thirty-four patients with NSCLC resistant to first- and second-generation EGFR-TKIs, who are positive for both act-EGFR and T790M in cfDNA at the time of progression, were enrolled in this study. Blood samples were collected at baseline and 3 months after osimertinib treatment. cfDNA was analyzed by droplet digital PCR, and the results were expressed as mutant allele frequency (MAF). At baseline, the MAF of act-EGFR was significantly higher than that of T790M (*p* < 0.0001). The act-EGFR MAF and ratio of T790M/act-EGFR MAF was significantly correlated with disease response (*p* = 0.02). The cutoff value of act-EGFR MAF and T790M/act-EGFR ratio was found to be 2.6% and 0.22, respectively. The PFS of patients with act-EGFR MAF of >2.6 and <2.6%, was 10 months vs. not reached, respectively (*p* =0.03), whereas patients with T790M/act-EGFR ≤ 0.22 had poorer PFS than those >0.22 (6 months vs. not reached, respectively, *p* = 0.01). act-EGFR MAF and T790M/act-EGFR MAF ratio are potential indicators of outcome in patients treated with osimertinib. The amount of activating EGFR mutations in circulating cfDNA is an indicator for monitoring osimertinib response (Del Re et al., 2018).

Mutation of tumor genes can be indicators of treatment response to immune checkpoint inhibitors (ICI) (Yuan et al., 2019). The study of Guibert et al. showed that sequencing of blood cfDNA can predict response to PD1 inhibitors in advanced NSCLC. The presence of a PTEN or STK11 mutation was correlated with early progression, while transversion mutations (Tv) in KRAS and TP53 predicted better outcomes. Patients with a low immune score (driver gene and/or PTEN or STK11 mutation and/or without KRAS or TP53 Tv) have a poor outcome with a median PFS of 2 months, compared with patients who have a high immune score (no driver gene, no PTEN or STK11, and with KRAS or TP53 Tv) and have a median PFS of 14 months (*p* = 0.0001, HR = 2.96). Another study showed that the change of allele fraction (AF) of ctDNA is correlated with clinical outcomes in 65 specimens, where the PFS is 14 months if AF decreases vs. 2 months if AF increases (*p* < 0.0001) (Guibert et al., 2019). Almodovar et al. (2018) demonstrated that longitudinal cfDNA analysis in patients with small cell lung cancer (SCLC) can reveal insights into treatment efficacy and disease relapse.

### Colorectal Cancer

Lyskjær et al. suggested that the level of cfDNA and ctDNA is correlated with the outcome of FOLFIRI treatment in metastatic colorectal cancer (mCRC). In their study, 24 patients with mCRC were enrolled and treated with FOLFIRI-based therapy. Blood was sampled before treatment, at days 7, 14, 21, and 60 after treatment and at progression, and the level of cfDNA and ctDNA was analyzed. Patients with a high level of pretreatment ctDNA or cfDNA (≥75th percentile) had significantly shorter PFS than those with a low level of ctDNA or cfDNA. Despite an overall decline in ctDNA level from pretreatment to first CT scan, 7 patients were identified with temporary increases in ctDNA and these patients had shorter PFS and OS, which was coincident with growth of drug-resistant cells. This study indicated that increased level of ctDNA in the first cycle of FOLFIRI treatment was an indicator of progressive disease and poor survival. Therefore, monitoring the level of ctDNA as early as 1 week after treatment is important for early detection of treatment failure (Lyskjaer et al., 2019).

Zitt et al. (2008) reported that the blood level of cfDNA decreased in patients who responded to chemoradiotherapy (responders, stage I–II), while the blood level of cfDNA increased in the nonresponders. The integrity index of cfDNA was lower after chemoradiotherapy (CRT) in responders when compared to nonresponders. In general, patients with CRC have 10 times higher integrity index than healthy individuals (Agostini et al., 2011). Sun et al. (2014) observed that the incidence of KRAS mutation at codon 12 in cfDNA decreased in responders.

Methylation test in liquid biopsy can be used in the absence of specific mutations of patients to monitor dynamic tumor burden. The selected biomarkers allowed monitoring tumor burden under different treatment regimens. Methylation test might be used to assess pharmacodynamics in clinical trials or in complementing conventional imaging analysis. Barault et al. (2018) reported that dynamics of methylation biomarkers was correlated with subjective tumor response and progression-free survival in patients with metastatic colorectal cancer.

### Prostate Cancer

cfDNA analysis can be used as a useful tool for precision medicine in castration-resistant prostate cancer (CRpC). Sumiyoshi et al. (2019) analyzed the cfDNA from 41 patients with CRpC. Most AR aberrations at baseline diminished with effective treatments, whereas AR amplification or mutations emerged in some patients with disease progression.

Goodall et al. reported that cfDNA analysis could guide the treatment of using poly(ADP)-ribose polymerase (PARP) inhibitor olaparib in metastatic prostate cancer (mPC). A decrease in blood level of cfDNA is correlated with the treatment outcome of PARP inhibitor olaparib in the Phase II Trial of Olaparib in Patients with Advanced Castration Resistant Prostate Cancer (Goodall et al., 2017). Briefly, somatic mutations of DNA repair were detectable in cfDNA in tumor tissues. The allele frequency of somatic mutations decreased selectively in responding patients (Chi-squared *p* < 0.001). Multiple subclonal aberrations revert somatic and germline mutations of DNA repair (BRCA2, PALB2) following response to olaparib treatment as disease progressed (Goodall et al., 2017).

To evaluate the tumor DNA fraction in urine cfDNA, Xia et al. developed an algorithm of Urine Genomic Abnormality (UGA) score which summed up the top 10 most significant segments with copy number changes. The UGA score is correlated with tumor burden, and the change in UGA score after stage-specific therapy reflected the status of disease progression and overall survival. The study demonstrated the potential clinical use of urine cfDNA in predicting treatment response and monitoring disease progression (Xia et al., 2016).

### Breast Cancer

Monitoring gene mutations is important in clinics. Notably, ESR1 mutation is detected at a high frequency in cfDNA of ER-positive metastatic breast cancer (MBC) and rarely found at the early-stage cancer. ESR1 mutation is enriched at disease progression, suggesting a role of ESR1 in MBC (Beije et al., 2018). The presence of ESR1 mutation indicates the development of endocrine resistance, especially resistant to aromatase inhibitors (Spoerke et al., 2016). Beije et al. (2018) suggested that ESR1 mutation is more prevalent in tumor progression (42%) than before progression (11%) (*P* = 0.04).

### Cervical Cancer

Tian et al. applied blood cfDNA analysis to evaluate the dynamic mutational change in 48 cancer driver genes in cervical cancer patients. They found that different treatments, including radiotherapy (*n* = 14), chemotherapy (*n* = 22), and surgery (*n* = 15), resulted in a significant decrease in the value of allele fraction deviation (AFD) (Wilcoxon, *p* = 0.029). The decrease of cfDNA AFD value was associated with reduced size of tumor in most patients. Progressive disease (metastasis) was detected in a subgroup of patients whose cfDNA AFD value did not reflect a reduction in tumor size. Also, a low AFD value at diagnosis followed by a later increased AFD value predicted disease relapse (Tian et al., 2019).

HPV ccfDNA could be used to select patients for HPV-type-specific T-cell-based immunotherapies. It might also have a value for evaluating antitumor activity of therapeutic drugs and long-term follow-up in patients with cervical cancer. Kang et al. proposed an approach to genotype and quantify HPV-circulating DNA in patients with HPV16- or HPV18-positive metastatic cervical cancer for potential disease monitoring and treatment decision making. In this retrospective study, HPV ccfDNA was detected in 100% (19 of 19) patients with HPV-positive metastatic cervical cancer but not in any of the 45 healthy blood donors. The HPV genotype harbored in the patients' tumors was correctly identified in 100% (87 of 87) of serial blood samples of nine patients who received TIL immunotherapy. In three patients who experienced objective cancer regression after TIL treatment, a transient HPV ccfDNA peak was observed 2–3 days after TIL infusion. Moreover, persistent clearance of HPV ccfDNA was found in two patients who experienced complete response (CR) after TIL immunotherapy (Kang et al., 2017).

### Bladder Carcinoma

Using Ultra-Deep Sequencing of Plasma Cell-Free DNA, Emil Christensen and his group evaluated early metastatic relapse and examined treatment efficacy of urothelial bladder carcinoma. They found that ctDNA positivity before or in the course of treatment indicated high-risk patients. The dynamic change of ctDNA is correlated with tumor recurrence (Christensen et al., 2019).

### Pancreatic Cancer

Analysis of cfDNA can be applied to evaluate the mutational makeup of cancer lesions and monitor cancer progression at the molecular level with no need of invasively acquired tissues from primary or metastatic lesions. Vietsch et al. showed that incorporation of cfDNA analysis provides crucial insights into the molecular change of progression of colon and pancreatic cancer. They revealed that cfDNA collected at the time of progression harbored 3–5 new mutations not detected in cfDNA collected at the earlier time points (Vietsch et al., 2017).

### Esophageal Cancer

Pasternack et al. (2018) found that detection of somatically altered cfDNA in patients with esophageal carcinoma in early stage is associated with postsurgical tumor recurrence.

## cfDNA as a Prognostic Biomarker for Cancer

cfDNA is attracting attention as a novel biomarker for predicting outcome in oncology and is able to predict overall survival in cancer patients.

### Breast Cancer

Panagopoulou et al. revealed that elevated concentrations of blood cfDNA are correlated with nonresponse to pharmacotherapy, shorter progression-free survival (PFS), and increased incidence of death in metastatic breast cancer (MBC). The methylation of WNT5A was significantly correlated with large tumor size, poor prognosis, and advanced-stage disease with short overall survival in MBC patients (Panagopoulou et al., 2019). Hussein et al. (2019) suggested that both plasma ALU-247 and ALU-115 repeats were preoperative prognostic biomarkers for breast cancer. Schiavon et al. (2015) showed that metastatic breast cancer patients with ESR1 mutations in their ctDNA had substantially shorter PFS on subsequent aromatase inhibitor-based therapy. Cheng et al. (2018) found that the integrity and blood level of cfDNA can serve as attractive and independent prognostic biomarkers for MBC patients at baseline and in the course of systematic therapy. The cfDNA mutation has also been evaluated in MBC. Chandarlapaty et al. (2016) showed that ESR1 mutation was associated with poor outcome in patients with metastatic breast cancer who were previously treated with an aromatase inhibitor.

### Lung Cancer

A prospective phase II trial evaluated cfDNA in patients with EGFR-mutant NSCLC treated with erlotinib until progression. Results indicated that the level of cfDNA is correlated with PFS. High level of cfDNA was defined as >55 EGFR mutation copies/mL compared with low level of cfDNA, which is <55 EGFR-mutant copies/mL. The median PFS of patients with high-level cfDNA (*n* = 18) was 9.3 months (95% CI, 6.3–14.8) vs. 14.0 months (95% CI, 9.2–20.1) for patients with low-level cfDNA (*n* = 41; *P* = 0.08) (Yanagita et al., 2016). Circulating cfDNA can be a predictive and prognostic biomarker in NSCLC. Several studies have assessed the predictive and prognostic value of cfDNA in NSCLC. A meta-analysis result demonstrated that NSCLC patients with high level of cfDNA were significantly associated with poor PFS. In addition, NSCLC patients who harbored EGFR mutation in cfDNA had a greater chance of response to EGFR-TKIs (Ai et al., 2016).

### Hepatocellular Carcinoma

Li et al. reported that cfDNA of methylated insulin-like growth factor-binding protein 7 (IGFBP7) was associated with overall survival and early tumor recurrence. Therefore, IGFBP7 could be an independent prognostic factor in hepatitis B virus-associated hepatocellular carcinoma after hepatectomy (Li et al., 2018). Ren et al. investigated the association between blood cfDNA level and overall survival and disease-free survival in patients with stage I–IV HCC. They found that a high level of cfDNA was associated with poor overall survival (Ren et al., 2006).

### Pancreatic Cancer

It was suggested that ctDNAs are shorter than cfDNA fragments originated from nonmalignant cells. Lapin et al. evaluated whether the size of cfDNA fragment and level of cfDNA had prognostic value in patients with advanced pancreatic cancer. Results indicated that both high cfDNA level and a cfDNA fragment size of ≤167 bp before treatment were associated with shorter PFS and OS (Lapin et al., 2018). Chen et al. suggested that cfDNA was a prognostic factor for OS and PFS in patients with pancreatic cancer. The presence of ctDNA, high level of cfDNA, mutation including Kras, ERBB2-exon17, and KrasG12V; and hypermethylation were associated with poor survival in pancreatic cancer (Chen et al., 2018).

### Colorectal Cancer

The analysis of cfDNA before surgery can indicate prognosis, and analysis of cfDNA after surgery can predict recurrence. A high level of blood cfDNA in the pretreatment phase is significantly correlated with poor survival (Schwarzenbach et al., 2008; Spindler et al., 2015). A prospective multicenter phase III trial indicated that the level of cfDNA can be a prognostic biomarker for oxaliplatin-based chemotherapy in metastatic colorectal cancer. A high level of cfDNA was associated with poor outcome. The median PFS is 7.7 months for the cfDNA level above the upper limit of normal (ULN) and 8.3 months for the cfDNA level below ULN. The median OS is 16.6 months for a cfDNA level above ULN and 25.9 months for a cfDNA level below ULN (Hamfjord et al., 2019). Patients with locally advanced-stage III–IV rectal cancer who had a high baseline cfDNA level showed short DFS (Schou et al., 2018).

The presence of methylation in HLTF and HPP1 genes in cfDNA in patients with stage I–IV colorectal cancer was associated with a poor OS (Wallner et al., 2006). Promoter methylation of three genes (HPP1, HLTF, and hMLH1) in blood cfDNA was positively correlated with tumor size. Also, methylation of HLTF and HPP1 genes was detected more frequently in metastatic CRC patients and in patients with high tumor stage (Philipp et al., 2012). The presence of a high level of TAC1 methylation 6–12 months after diagnosis was associated with early recurrence (Tham et al., 2014). The methylated status of the APC gene in cfDNA was associated with higher stage and older age. Moreover, patients with the unmethylated promoter of APC or RASSF1A genes showed better OS than patients with promoter hypermethylation (Matthaios et al., 2016).

### Oral Squamous Cell Carcinoma

Lin et al. evaluated plasma cfDNA in 121 patients with oral squamous cell carcinoma (OSCC) and 50 matched controls using quantitative spectrometry. Results indicated that blood cfDNA was significantly increased in patients with OSCC compared to controls. The high level of blood cfDNA was correlated with large tumor size, cervical lymph node metastasis, and late stage. High level of blood cfDNA was associated with a poor prognosis of OSCC (Lin et al., 2018).

### Prostate Cancer

Epigenetic biomarkers in circulating cfDNA can predict survival of castration-resistant prostate cancer patients. A high level of cfDNA and methylated GSTP1 and APC was observed in CRPC patients compared to healthy subjects. Moreover, the baseline level of cfDNA and methylated APC and GSTP1 before treatment can predict overall survival (Hendriks et al., 2018).

### Melanoma

Valpione et al. (2018) revealed that the blood total cfDNA is a surrogate biomarker for tumor burden and can predict overall survival in patients with metastatic melanoma.

## Conclusion

Analysis of cfDNA in liquid biopsy is a minimally invasive, low-cost, and promising alternative to tumor biopsy. Liquid biopsy based on cfDNA can not only detect cancer recurrence more quickly than the current radiological imaging technology but also provide insights on the molecular evolution of minimal residual disease (MRD) in tumor progression through molecular characterization of cfDNA, which is of great significance for the prevention and treatment of tumor recurrence. The most common body fluid is blood. In addition, different body fluids will be selected for different detection targets. For example, saliva is often used in the detection of head and neck tumor, and cerebrospinal fluid is applied in the diagnosis of tumors of the central nervous system, urine in the case of urinary tract cancers, and pleural effusion for respiratory tract cancers. However, due to the very low concentration of cfDNA in blood or other samples, it has high requirements for sensitivity and detection limit of the detection method. The gold standard of cfDNA analysis, including quantitative PCR (qPCR) and digital PCR, is becoming mature. In recent years, the development of whole-genome sequencing and novel PCR-free biosensing approaches have also made some breakthroughs. In this article, we reviewed studies exploring cfDNA for diagnostic, therapeutic, and prognostic evaluation in various types of cancers. This study supports the idea that cfDNA analysis for cancer represents a potential research area and will have wide application in clinics.

## Author Contributions

Y-yY, Q-rG, and J-yZ conceived the review. Y-yY, F-hW, and Z-yZ searched the literature and drafted the manuscript. RA, H-yZ, and W-mZ critically appraised the literature. HY and J-qL edited the manuscript. All authors approved the final version of the manuscript.

## Conflict of Interest

The authors declare that the research was conducted in the absence of any commercial or financial relationships that could be construed as a potential conflict of interest.
